# Novel probiotics adsorbing and excreting microplastics *in vivo* show potential gut health benefits

**DOI:** 10.3389/fmicb.2024.1522794

**Published:** 2025-01-10

**Authors:** Xin Teng, Tengxun Zhang, Chitong Rao

**Affiliations:** Bluepha Co., Ltd., Shanghai, China

**Keywords:** microplastics, probiotics, *Lactobacillus*, adsorption, MP-associated health risks, high-throughput screening

## Abstract

Microplastics (MP) contamination in food and water poses significant health risks. While microbes that form biofilm show potential for removing MP from the environment, no methods currently exist to eliminate these non-degradable MP from the human body. In this study, we propose using probiotics to adsorb and remove ingested MP within the gut. We conducted a comprehensive evaluation of 784 bacterial strains to assess their ability to adsorb 0.1 μm polystyrene particles using a high-throughput screening method. Among the tested strains, *Lacticaseibacillus paracasei* DT66 and *Lactiplantibacillus plantarum* DT88 exhibited optimal adsorption *in vitro* and were effective across various MP types. In an animal model, mice treated with these probiotics demonstrated a 34% increase in PS excretion rates and a 67% reduction in residual polystyrene (PS) particles within the intestine. Additionally, administration of *Lactiplantibacillus plantarum* DT88 mitigated PS-induced intestinal inflammation. Together, our findings demonstrate a novel probiotic strategy for addressing MP-associated health risks, emphasizing the potential of strain-specific probiotics to remove MP from the gut environment.

## 1 Introduction

Plastics are widely used for food packaging, healthcare, construction materials, furniture, textiles, and so on. The consumption of these synthetic polymers is growing rapidly due to their versatility and low production costs. However, approximately 80% of manufactured plastics are improperly processed, resulting in plastic pollution in the natural environment (Geyer et al., 2017). Under physical, chemical, and biological pressures, plastics are gradually disintegrated into smaller debris less than 5 mm in length, known as microplastics (MP) (Osman et al., 2023). Over time, MPs have dispersed into soil, water, atmosphere, and even living creatures (Osman et al., 2023), raising alarm about the long-term risks of these tiny pollutants to both ecosystems and human health (Landrigan et al., 2023; Rose et al., 2023; Lozano et al., 2024).

Microplastic contamination has been found in daily food and drinking water and enters the human body through ingestion (Li et al., 2023; Sun and Wang, 2023). It is reported that up to 5 g of MPs could be consumed per week (Senathirajah et al., 2021). The ingested MPs can be translocated across the gastrointestinal mucosa and deposit in biological fluids and organs (Powell et al., 2010; Sun and Wang, 2023; Yang et al., 2023). Given their inert chemical property, MPs cannot be metabolized and degraded inside the human body. Cytotoxicity and inflammation represent the main adverse health effects (Xu et al., 2022). Increasing evidence has linked MP exposure to detrimental health effects including neurotoxicity, cardiovascular disease, and others (Prata et al., 2020), possibly through MP-mediated oxidative stress, DNA damage, metabolic disorder and apoptosis (Li et al., 2023; Sun and Wang, 2023). For example, a recent landmark study showed in a three-year cohort that people who had MPs lodged in carotid artery were more likely to experience heart attack (Marfella et al., 2024).

Probiotics, living microbial supplements consumed by the host, have demonstrated the ability to protect against health damage caused by toxic materials such as heavy metals and plasticizers. They achieve this protective effect by either binding to or degrading these toxins(Zhao et al., 2013; Lili et al., 2017; Ju et al., 2019; Alcántara et al., 2020; Huang et al., 2020; Yang and Pei, 2020; Baralić et al., 2021; Kyrila et al., 2021). The use of probiotics has shown a great potential for their effectiveness and biocompatibility for trapping and reducing MP in natural seawater (Liu et al., 2021). Bacteria can form biofilms, sticky matrices formed that aggregates and sediments the MPs through hydrophobic binding to the MP surface (He et al., 2022; Romero et al., 2022; Zhao et al., 2023). Despite their beneficial effects in countering MP-induced damage by improving the microbiome and modulating the host immune system (Chen et al., 2022; Li et al., 2022; Zhang et al., 2023; Yu et al., 2024), and by reducing oxidative stress through MP binding (Dong et al., 2022; Athulya et al., 2024), few approaches have been reported for removing ingested MP from the human body.

In this study, we sought to explore the potential application of probiotic bacteria in removing ingested MPs from the gut. We first screened for bacterial strains with high MP-adsorbing capacity. Candidate probiotics were subjected to further *in vivo* validation for MP elimination in MP-ingested mice. We further investigated how these probiotics altered MP-induced inflammatory response. These results shed light on using probiotics as a new strategy to reduce the health risks of MP exposure.

## 2 Materials and methods

### 2.1 Microplastics

Fluorescent polystyrene (PS) particles were purchased from the Baseline Chromatography Technology Development Center (Tianjin, China). Polyethylene (PE), polycarbonate (PC), polypropylene (PP) and polyethylene terephthalate (PET) particles were obtained from BASF (China) Company Limited. Particle size was characterized by Topsizer Plus Laser Particle Size Analyzer with SCF-126B micro-circulating sampler (Omec, China).

### 2.2 High-throughput screening of microplastic adsorbing bacterial strains *in vitro*

Fermented foods were diluted with sterile PBS and then plated on GAM (Gifu Anaerobic Medium), BBL™ and MRS (de Man, Rogosa, and Sharpe) agar plates. The plates were incubated under anaerobic condition at 37°C for 48 h. Single colonies were streaked into corresponding fresh medium and incubated under anaerobic condition for another 24 h. After incubation, the bacteria were harvested by centrifugation, washed twice with sterile PBS solution, and resuspended to 1 × 10^9^ CFU/mL in PBS solution.

20 μL of 1 × 10^9^ CFU/mL bacterial suspension was added into a 96-well plate in which each well contained 180 μL of 0.16 mg/mL 0.1 μm PS fluorescent particle working solution. The 96-well plate was placed on shaker and incubated for 4 h in the dark at 37°C, 800 rpm. The solution was then centrifuged at 2000 rpm for 10 min, and the fluorescence intensity of the supernatant was measured using a Tecan Microplate Reader (excitation 488 nm, emission 518 nm). The adsorption ratio (AR) was calculated as follows: AR (%) = (A1−A2)/A1 × 100%, where A1 and A2 represent the fluorescence intensity of the supernatant incubated with the PBS control, and candidate bacteria, respectively. Samples were prepared in triplicates. The taxonomy of selected strains was determined by 16S rRNA Sanger sequencing.

### 2.3 Observation of the adsorption of microplastics *in vitro*

Selected *Lactobacillus* strains were inoculated into MRS medium and anaerobically cultured at 37°C for 24 h. After culturing, the bacterial solution was centrifuged at 4000 rpm for 10 min. The supernatant was discarded, and the pellet was washed twice by adding 450 μL of sterile PBS buffer. Next, PBS was added to resuspend the bacterial solution and adjust its concentration to 1 × 10^9^ CFU/mL. Subsequently, 100 μL of the bacterial suspension was transferred into a 1.5-mL EP tube, and 900 μL of sterile PBS or PS fluorescent particle working solution (0.16 mg/mL, particle size 0.1 μm) was mixed. The mixture was then shaken in the dark for 4 h at 37°C and 800 rpm. At the end of the incubation period, the solution was examined for bacteria-PS particle aggregates. Finally, the incubation solution was centrifuged at 2,000 rpm for 10 min to observe the color of the bacterial precipitate.

### 2.4 Tolerance to gastric acid and bile acid *in vitro*

The overnight culture of isolated strains was collected by centrifugation at 4,000 rpm for 10 min. The precipitate was washed with PBS solution and resuspended with MRS medium at pH 2.5 or MRS medium containing 0.1% bile salt. Bacterial suspension was then incubated under anaerobic condition at 37°C for 3 h. *Lactobacillus rhamnosus* GG was used as a control. The survival rate was calculated as follows: Survival rate (%) = C1/C0 × 100%, where C0 and C1 represent the number of viable cells in the culture before and after the incubation, respectively. Samples were prepared in triplicates.

### 2.5 Determination of antioxidant activity *in vitro*

The antioxidant activity was determined by 2,2-Diphenyl-1-picrylhydrazyl (DPPH) radical-scavenging capacity according to the previous method (Li et al., 2012). Briefly, *Lactobacillus* was suspended in PBS solution at a concentration of 1 × 10^9^ CFU/mL. A 3 μg/mL vitamin C (Vc) solution was used as positive control and PBS as negative control. 500 μL of the bacterial suspension was added to 500 μL of 0.2 mmol/L DPPH ethanol solution. The reaction of bacterial suspension and ethanol was used as blank. Following thorough mixing, the reaction was placed on a shaker at 30°C, 800 rpm for 30 min. The absorbance of 100 μL of supernatant was measured at 517 nm after centrifugation at 4,000 rpm for 10 min. The DPPH radical scavenging activity was calculated as follows: Scavenging activity (%) = (1 – (A1 – A0)/Ac) × 100%, where A1 represents the absorbance value in the experiment group, A0 represents the absorbance value in the no-DPPH blank, and Ac represents the absorbance value in the PBS-treated negative control. Samples were prepared in triplicates.

### 2.6 Scanning electron microscopy (SEM)

MP particles were resuspended in a PBS solution containing 0.1% Tween-80. 1 × 10^8^ CFU of bacteria and 0.144 mg MP were mixed in a solution, placed on a shaker and incubated for 4 h at 37°C, 800 rpm. After incubation, the precipitate was harvested by centrifugation at 4000 rpm for 10 min and subjected to SEM imaging (TESCAN MIRA LMS, Czechia).

### 2.7 PS excretion rate study *in vivo*

Six-week-old male SPF C57 mice were purchased from Gempharmatech Co., Ltd. (Nanjing, China). Mouse care and all experimental procedures were approved by the Animal Ethics Committee of HuaYuan Shidai Biotech Co., Ltd. (Beijing, China) according to institutional animal ethics guidelines (Permission No. HYSD2022-10). All animals were housed under conditions with a room temperature of 22°C ± 2°C and humidity of 50% ± 10%.

Fourty six-week-old male SPF C57 mice were equally divided into 4 groups and were administered with 1 × 10^9^ CFU of probiotics or equal volume of saline daily by oral gavage for 7 days. Following the final gavage, mice were fasted for 16 h, then given a single oral gavage with 100 μL of 10 mg/mL 5 μm PS solution. After 20 min of PS gavage, mouse intestine was dissected from the cardia to the cecum. The fluorescence position of PS particles was photographed using IVIS® Lumina III *In Vivo* Imaging System (PerkinElmer, USA). Images were processed by ImageJ software. Excretion rate (%) = L1/L0 was calculated, where L1 represents the movement length of fluorescent PS particles in the intestine, and L0 represents the total length of the intestine from pylorus to cecum.

### 2.8 Determination of residual PS in the intestinal tract after PS exposure *in vivo*

Six-week-old male SPF C57 mice were purchased from BesTest BioTech Co., Ltd. (Zhuhai, China) and acclimatized for 1 week. Mouse care and all experimental procedures were approved by the Animal Ethics Committee of TOP Biotech Co., Ltd. (Shenzhen, China) according to institutional animal ethics guidelines (Permission No. TOPGM-IACUC-2023-0057). All animals were housed under conditions with a room temperature of 22°C ± 2°C and humidity of 50% ± 10%.

Fifty six-week-old male SPF C57 mice were equally divided into five groups and were treated with 100 μL of 10 mg/mL 5 μm PS solution by intragastric administration for 7 days to establish PS exposure model (Jin et al., 2021; Zolotova et al., 2022; Wang et al., 2023). Three groups were orally gavaged with 1 × 10^9^ CFU probiotics in the morning and exposed to fluorescent PS particles in the afternoon. In the control group, mice were given an equal volume of saline in the morning and exposed to PS particles in the afternoon. In the blank group, mice were given saline twice every day. Following the final PS particles gavage, mice were fasted for 16 h. Mice were anesthetized and sacrificed. Blood, ileum and cecum samples were collected for subsequent analysis.

The amount of fluorescent PS particles in the intestine and feces was determined using flow cytometry referring to previous studies (Nielsen et al., 2014; Jin et al., 2021; Liang et al., 2021). Feces and freshly collected ileum and cecum samples were weighed and homogenized in 400 μL of digestion solution (1 g/L proteinase K, 5 g/L SDS, 23 g/L Na_2_HPO_4_, and 4.6 g/L NaH_2_PO_4_). After incubation at 37°C for 12 h, the homogenate was diluted with PBS buffer, and then filtered through a 100 μm cell strainer.

The resulting samples were subjected to flow cytometry analysis using NovoCyte 2100YB Flow Cytometers (Agilent, United States). The minimum threshold of FITC-H was set at 2.0 × 10^6^ and the minimum threshold of FSC-H was set at 60000 to exclude background noise in samples. Collected data were analyzed with FlowJo (v10.8.1) software and the amount of PS particles was determined using a standard curve.

### 2.9 ELISA analysis of intestinal inflammation biomarkers

The intestinal tissues were harvested as described above. Concentrations of IL-10, IL-6, TNF-*α* and IL-1β in the intestinal tissues were determined by respective ELISA kits (Meimian, China) according to the manufacturer’s protocol.

### 2.10 Short chain fatty acid (SCFA) analysis of fecal samples

Fecal samples taken before the final gavage from mice, as described in Materials and Methods 2.7 were weighed and stored in methanol containing 5 mg/kg sodium acetate-D4 (Sigma Aldrich). The samples were homogenized with 20 min ultrasonication. After the esterification process, the SCFA concentration was analyzed with gas chromatography coupled to mass spectrometry (GC–MS).

### 2.11 Statistics

Statistical differences between two groups were determined using two-tailed Student’s t-test. Differences between more than two groups were tested using one-way analysis of variance (ANOVA). Statistical analysis was performed using GraphPad Prism v8.0. *p*-value <0.05 was considered statistically significant.

## 3 Results

### 3.1 *In vitro* screening probiotics for MP adsorption

**Figure 1:**
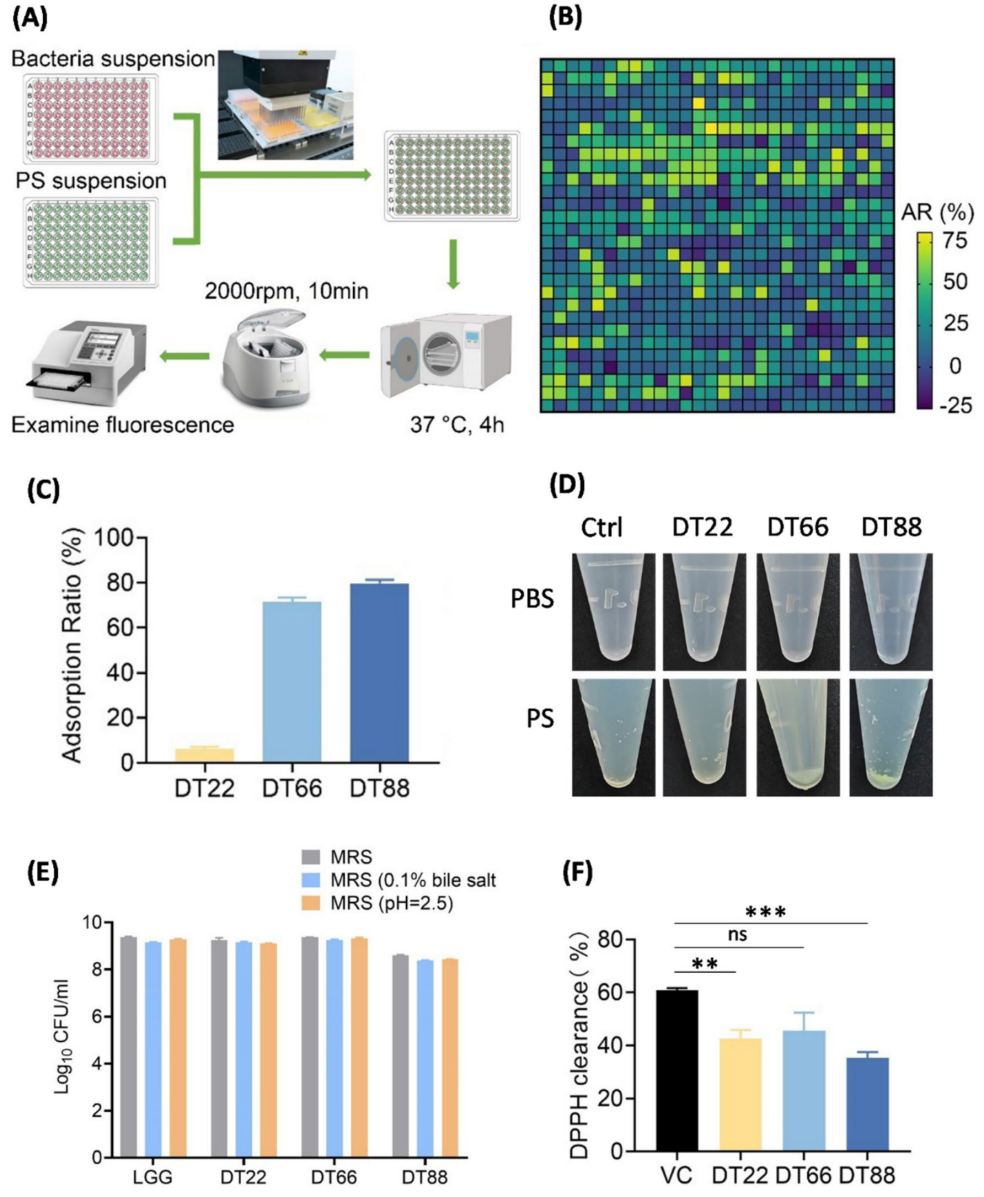
Screening probiotic strains with high microplastics adsorption. **(A)** Flowchart of the high-throughput screening of MP adsorption. **(B)** Heatmap of MP adsorption ratio (AR) of 784 strains. **(C)** AR of three selected bacterial strains. Data are shown as means ± SEM. **(D)** *Lacticaseibacillus paracasei* DT66 and *Lactiplantibacillus plantarum* DT88 formed flocculent precipitate after co-incubation with 0.1 μm PS fluorescent particles. **(E)** Number of viable cells after incubation with MRS, MRS with 0.1% bile salt, or MRS at pH 2.5 for 2 h. Data are shown as means ± SEM. **(F)** Antioxidant activities of the selected three strains, calculated as DPPH scavenging activity. Data are shown as means ± SEM, *n* = 3. ns not significant, ** *p* < 0.01, *** *p* < 0.001.

784 probiotic strains from fermented foods were isolated and served as candidate bacteria (see Methods). We implemented a high-throughput screening method with fluorescein isothiocyanate (FITC)-labeled PS particles to screen for bacterial strains with high MP adsorption capacity (Figure 1A). The PS fluorescence intensity in solution decreases upon bacteria adsorbing and precipitating these particles after incubation and centrifugation. Using the relative fluorescence intensity decrease, i.e., adsorption ratio (AR), we found that 87 strains had an AR greater than 60%, with the highest AR being 80.5% (Figure 1B).

Three probiotic strains with high or low AR values were selected for further analysis. Two strains designated as *Lacticaseibacillus paracasei* DT66 and *Lactiplantibacillus plantarum* DT88 have AR values of 71.4 and 79.8%, respectively, while the other strain, *Lactiplantibacillus plantarum* DT22 has an AR value of 6.2% (Figure 1C). *Lacticaseibacillus paracasei* DT66 and *Lactiplantibacillus plantarum* DT88 co-aggregated and formed flocculent precipitate with PS particles after incubation, while there was minimal precipitate formed in the bacterial solution without PS particles (Figure 1D). After centrifugation, white precipitates were observed in bacterial solution without co-incubation with PS particles. The precipitate of *Lacticaseibacillus paracasei* DT66 and *Lactiplantibacillus plantarum* DT88 after co-incubation with PS particles turned green while *Lactiplantibacillus plantarum* DT22 remained white, demonstrated the different PS adsorbing capacity of selected *Lactobacillus* strains (Supplementary Figure S1).

**Figure 2:**
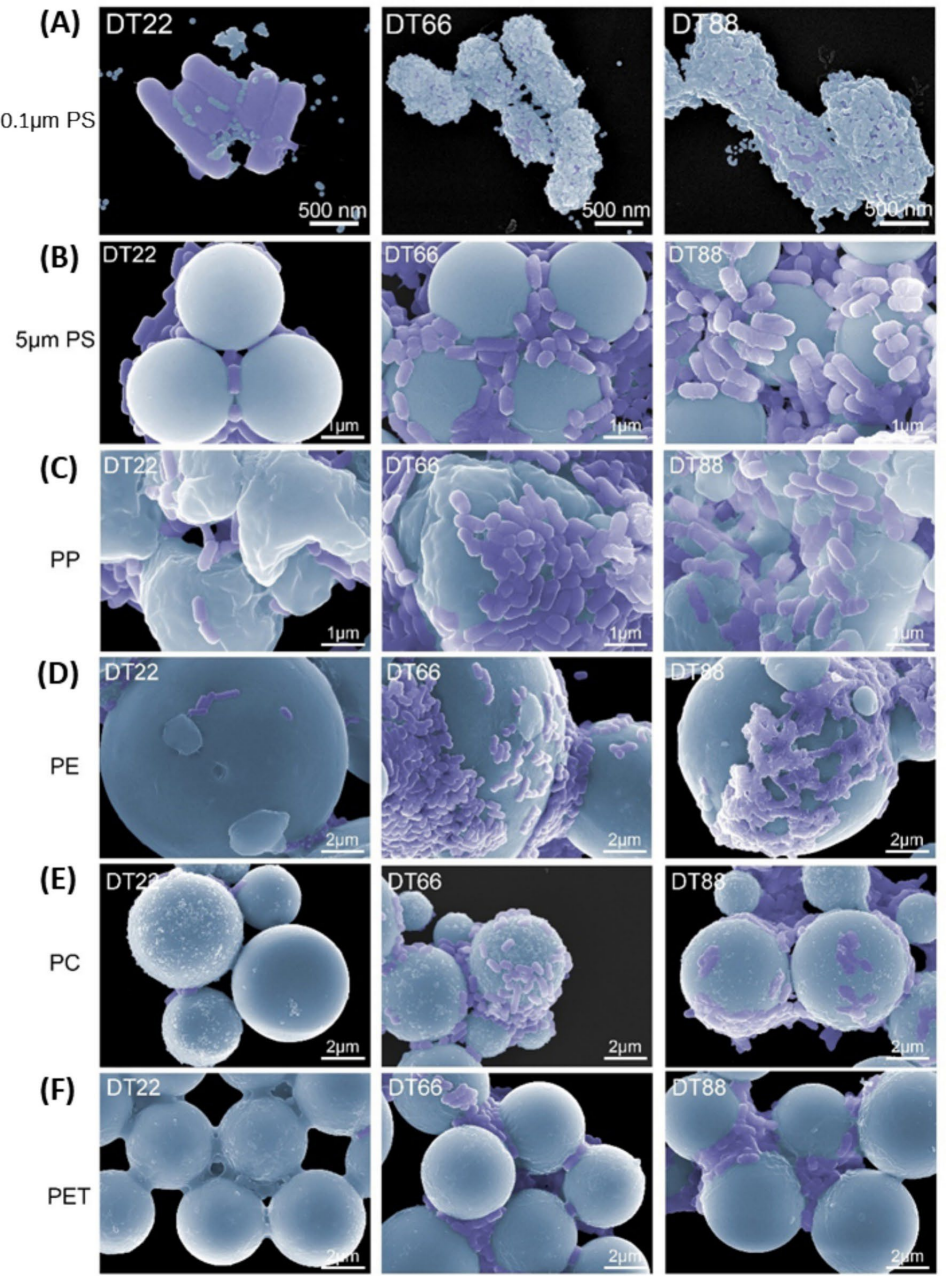
Scanning electron microscopy (SEM) images of bacteria aggregated with microplastics. Representative SEM images of **(A)** 0.1 μm PS, **(B)** 5 μm PS, **(C)** PP, **(D)** PE, **(E)** PC, **(F)** PET adsorbed with bacteria. For **(A)** magnification: 20,000×, scale bar: 500 nm; for **(B,C)** magnification: 10,000×, scale bar: 1 μm and for **(D–F)** magnification: 5,000×, scale bar: 2 μm. Bacteria were colorized purple. MP were colorized cyan.

To identify candidate probiotics with high application potential, we further validated for their tolerance to bile salt and gastric acid, as well as their antioxidant potentials. All three *Lactobacillus* strains demonstrated good tolerance for bile salts and gastric acids (Figure 1E), and showed good antioxidant potentials (Figure 1F). To confirm the screening results for MP adsorption, we subjected the three strains to SEM visualization after co-incubation of bacteria and PS particles (Figure 2). Indeed, *Lacticaseibacillus paracasei* DT66 and *Lactiplantibacillus plantarum* DT88 were fully covered by 0.1 μm PS particles, while much fewer PS particles adhered to the surface of *Lactiplantibacillus plantarum* DT22 (Figure 2A). Similarly, we observed extensive aggregation of *Lacticaseibacillus paracasei* DT66 and *Lactiplantibacillus plantarum* DT88 cells, but less *Lactiplantibacillus plantarum* DT22 cells, aggregated on the surface of 5 μm PS particles (Figure 2B).

In addition to PS particles, we further assessed these bacteria’s adsorption ability to other MP particles commonly found in the environment, including PE, PC, PP, and PET (Supplementary Figure S2). Aggregation of bacterial cells on the surface of different MP particles was observed for both *Lacticaseibacillus paracasei* DT66 and *Lactiplantibacillus plantarum* DT88, but much less for *Lactiplantibacillus plantarum* DT22 (Figures 2C–F).

Taken together, our high-throughput screening and multi-step confirmation resulted in two probiotic strains, *Lacticaseibacillus paracasei* DT66 and *Lactiplantibacillus plantarum* DT88, that exhibited high adsorption capacity on a variety of common MPs.

### 3.2 Probiotics accelerate MP excretion *in vivo*

**Figure 3:**
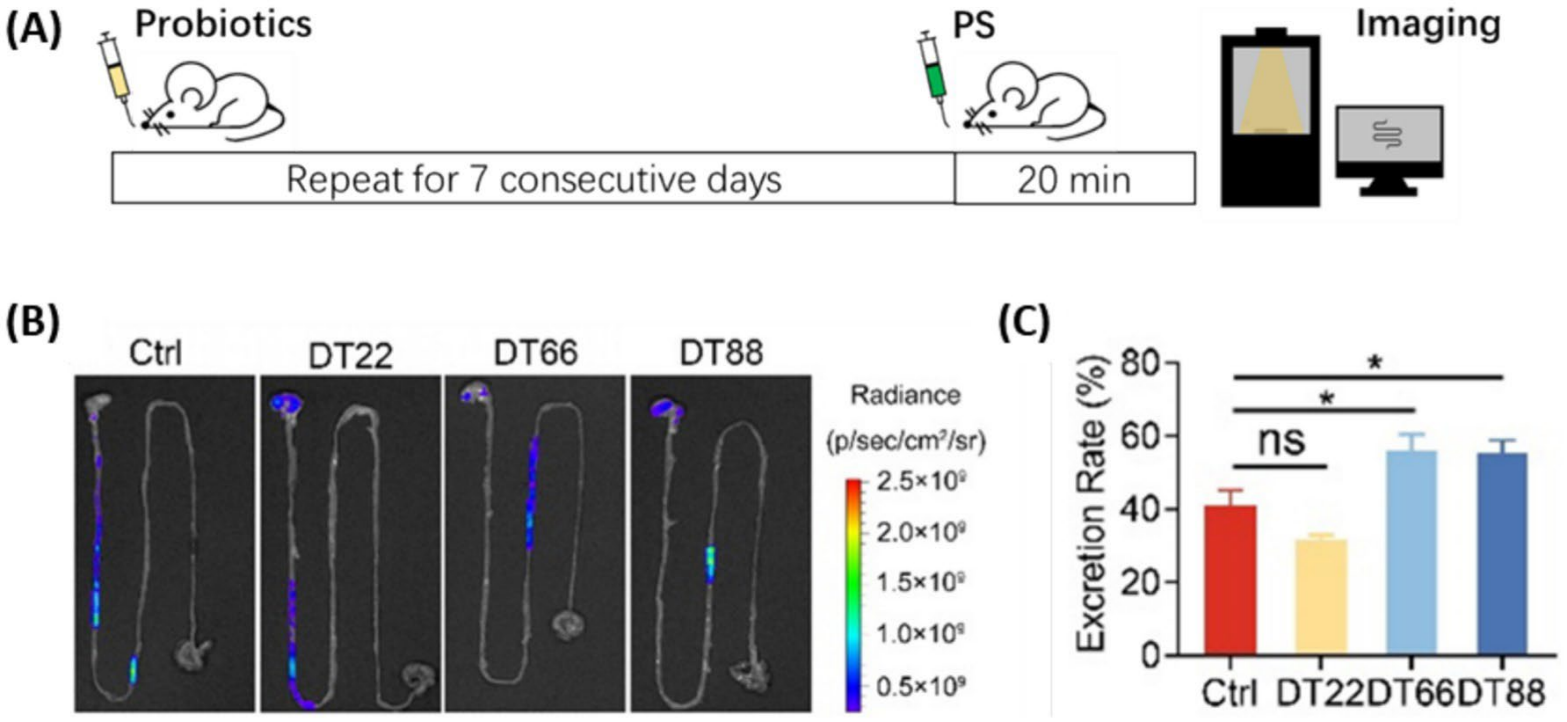
Probiotics administration promoted MP excretion. **(A)** Flowchart of the excretion rate study. **(B)** Representative fluorescent images of the intestinal tract from the cardia to the cecum. **(C)** Excretion rate of PS particles. Data are shown as means ± SEM, *n* = 10. ns not significant, * *p* < 0.05.

We hypothesize that the larger size of bacteria-MP aggregates may facilitate MP excretion from the gastrointestinal (GI) tract. We next investigated whether the administration of *Lacticaseibacillus paracasei* DT66 and *Lactiplantibacillus plantarum* DT88 could promote MP excretion in a mouse model. Monitoring the movement of gavaged material from the pylorus to the cecum can be used to evaluate short-time intestinal excretion rate *in vivo* (Zhou et al., 2022). Accordingly, we administered mice with each *Lactobacillus* strain or saline control for seven consecutive days, followed by one shot oral gavage of fluorescent PS particles to examine the immediate MP excretion rate (Figure 3A). Compared to the saline control and *Lactiplantibacillus plantarum* DT22 groups, mice treated with *Lacticaseibacillus paracasei* DT66 and *Lactiplantibacillus plantarum* DT88 showed the fluorescent PS moving further down the intestinal tract (Figure 3B). Quantification results showed that the average excretion rate of PS was 41.0% in the control group, while the number increased to 55.9 and 55.2% in the *Lacticaseibacillus paracasei* DT66 and *Lactiplantibacillus plantarum* DT88 groups, respectively (Figure 3C). This result indicated that these probiotics’ high MP adsorption capacity correlates with high MP excretion effect in mice.

Besides MP aggregation, we explored whether GI motility may also play a role in MP removal, as probiotics can produce SCFAs that regulate GI motility (Martin-Gallausiaux et al., 2021). The acetic acid and propionic acid levels in the feces did not show significant changes in all three probiotic treated groups compared to the saline-treated control after 7 days of probiotic administration, while the butyric acid level increased to different extents (Supplementary Figure S3). These results suggested that GI motility may also play a role in the acceleration of PS excretion.

### 3.3 Probiotics reduce MP retention *in vivo*

**Figure 4:**
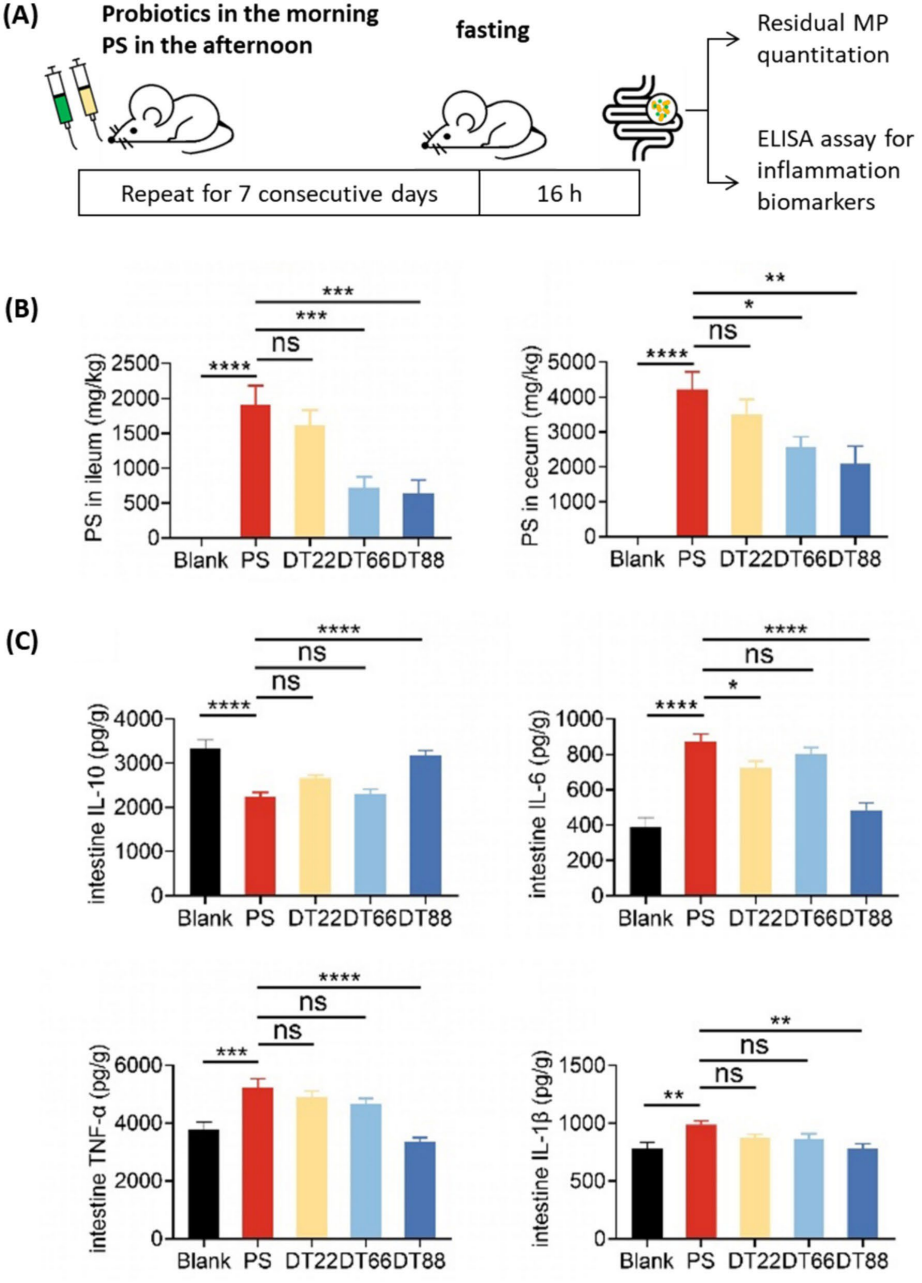
Probiotics administration reduced MP retention and alleviated MP-induced intestinal inflammation. **(A)** Flowchart of probiotics administration in PS-exposed mice. **(B)** Amount of PS remaining in ileum and cecum at 16 h after the last PS gavage. **(C)** Concentration of IL-10, IL-6, TNF-*α* and IL-1β in intestine. Data are shown as means ± SEM, *n* = 10. ns not significant, * *p* < 0.05, ** *p* < 0.01, *** *p* < 0.001, **** *p* < 0.0001.

With candidate *Lactobacillus* strains showing promising MP adsorption and excretion capacities, we next examined if MP accumulation and the subsequent immune response in vivo can be ameliorated by probiotic treatment. To this end, we orally gavaged mice with 1 × 10^9^ CFU bacterial strains or saline in the morning and 1 mg of fluorescent PS in the afternoon for seven consecutive days (Figure 4A).

Pilot time-series excretion experiment indicated that >99% of the PS in the feces were excreted at 16 h post the last PS gavage (Supplementary Figure S4). To determine the residual MP in the intestine, we collected the ileum and cecum samples at 16 h after the last PS gavage. For the ileum tissues, the PS particle amount was found reduced by 61.9 and 66.8% in mice treated with *Lacticaseibacillus paracasei* DT66 and *Lactiplantibacillus plantarum* DT88 compared to saline group, respectively (Figure 4B). A similar decrease by these two strains was also observed for the cecum samples (Figure 4B). The low-adsorbing strain *Lactiplantibacillus plantarum* DT22 did not change the residual PS levels in both ileum and cecum. Taken together, these results showed that *Lacticaseibacillus paracasei* DT66 and *Lactiplantibacillus plantarum* DT88 could effectively reduce MP retention in the intestinal tract.

### 3.4 Probiotics ameliorate MP-induced inflammation

MP exposure could trigger an immune response in the intestine (Liu et al., 2022). To evaluate the impact of PS removal on gut inflammation, we collected intestine tissue samples to examine the levels of different inflammatory biomarkers in mice, including the anti-inflammatory cytokine Interleukin 10 (IL-10) and pro-inflammatory cytokines IL-6, TNF-*α* and IL-1β. We also tested serum cytokine to assess systemic inflammation, in addition to intestinal cytokine levels which represent localized immune effects. We observed decreased IL-10 levels in the serum and ileum in the PS-treated mice compared to the blank group (Figure 4C, Supplementary Figure S5).

Administration of *Lacticaseibacillus paracasei* DT66 significantly alleviated the IL-10 reduction only in the serum, and treatment with *Lactiplantibacillus plantarum* DT88 significantly prevented the IL-10 reduction in both serum and ileum compared to the control group (Figure 4C, Supplementary Figure S5). With Regard to the pro-inflammatory cytokines, we found higher levels of IL-6, TNF-α and IL-1β in the ileum after PS exposure, and treatment with *Lactiplantibacillus plantarum* DT88 restored these cytokines to the blank group levels (Figure 4C). These results suggested that *Lactiplantibacillus plantarum* DT88 is an effective probiotic strain to ameliorate MP-induced inflammatory responses.

## 4 Discussion

Microplastics (MP) accumulate in terrestrial and marine environments, infiltrating the bodies of marine organisms and animals, ultimately contaminating the human food chain. Seafood is considered the primary source of MP ingestion, with estimated annual consumption per person ranging from 39,000 to 52,000 particles. Additionally, bottled water consumption contributes an extra 90,000 MP particles annually (Cox et al., 2019). The detection of MP in human stool indicates that approximately 200 MP particles are excreted daily (Schwabl et al., 2019). Therefore, these non-excreted MP particles in the human body can be transported into the bloodstream and accumulate in tissues (Leslie et al., 2022). There is increasing evidence demonstrating the toxic effects of MP exposure (Li et al., 2023). The toxicity of MP is determined by factors such as size, concentration, material, and surface charge. Smaller particles are more easily translocated into intestinal cells (Walczak et al., 2015). The mixture of MP of different sizes exacerbated the dysfunction of the intestinal barrier, which was caused by ROS-mediated epithelial cell apoptosis (Liang et al., 2021). A previous study found that PE particles may be more effective in crossing the intestinal barrier than PS particles when evaluating gastrointestinal uptake of microplastic particles (Stock et al., 2021). Therefore, the development of strategies to remove MPs from the human body is of paramount importance.

In our study, we explored the potential of probiotics as a novel strategy for the removal of ingested MPs from the human body. We successfully developed a high-throughput screening assay to determine the bacteria-MP adsorption capacity. We identified two probiotic strains, *Lacticaseibacillus paracasei* DT66 and *Lactiplantibacillus plantarum* DT88, with a high adsorption capacity to MP. *Lacticaseibacillus paracasei* DT66 and *Lactiplantibacillus plantarum* DT88 aggregated with MP, significantly increasing the excretion rate of MP, resulting in a reduction of more than 60% of remaining MP in the gut. *Lacticaseibacillus paracasei* and *Lactiplantibacillus plantarum* are commonly found in fermented foods, including dairy products and plant fermentations, as well as in human and animal gastrointestinal tracts. These strains are widely used as probiotics in the food industry and have received GRAS (generally recognized as safe) status from the US FDA (Food and Drug Administration) and QPS (qualified presumption of safety) status from EFSA (European Food Safety Authority) for use in processing foods and probiotic supplements (Ray and Joshi, 2014; EFSA Panel on Biological Hazards (BIOHAZ) et al., 2020).

The primary mechanism underlying the probiotic-mediated MP removal appears to be the co-aggregation between bacteria and MPs. Co-aggregation is a valuable property of probiotic bacteria for their antagonistic activity against pathogens and the clearance of toxic reagents (Sadeghi et al., 2022; Rashad Hameed and Abdul Sattar Salman, 2023). Extracellular polymeric substances (EPS), composed mainly of polysaccharides, proteins, nucleic acids, and lipids, act as ligands in the biosorption of heavy metals due to their abundance of negative charges (Yue et al., 2015). Previous studies have shown that plastic particles with positive charges are more toxic and have a higher uptake efficiency for cells (Roshanzadeh et al., 2020). Zhao’s research showed that the hydrophobic interaction involved in the aggregation of plastics on the surface of *Lactobacillus* (Zhao et al., 2023), thereby facilitating the aggregation and subsequent removal of MPs from the gastrointestinal tract.

Despite the promising results, there are several limitations in this study. First, although we demonstrated that *Lacticaseibacillus paracasei* DT66 and *Lactiplantibacillus plantarum* DT88 could effectively adsorb PS particles, we only quantified the adsorption ratios for PS particles and did not measure the AR of other types of MPs, such as PE, PC, PP, and PET. Different plastics possess diverse physicochemical properties that could influence their interaction with bacterial surfaces, including their hydrophobicity, electrostatic charge, and the compatibility of their surfaces with bacterial EPS. The diversity of plastic types in the environment necessitates a more comprehensive understanding of how probiotics interact with different MP materials.

Another limitation is the difficulty of characterizing probiotic-MP aggregates *in vivo*. The complex nature of the microbiome, the presence of food residues, and other intestinal contents in the gut makes it challenging to isolate and characterize the probiotic-MP aggregates accurately. Moreover, the fate of these aggregates in other organs, such as the liver or brain, remains unclear. Given that MPs can translocate from the gut into systemic circulation (Li et al., 2023), understanding the distribution of aggregated MPs throughout the body and their subsequent elimination is crucial. We hypothesize that the larger size of the bacteria-MP aggregates could reduce their ability to translocate across the gut barrier into the bloodstream and other organs (Walczak et al., 2015).

In addition to the mechanical effects of probiotic-MP aggregation, the impact of probiotics on gut motility and immune responses needs further exploration. Short-chain fatty acids (SCFAs), particularly butyrate, are produced by gut bacteria during fermentation of dietary fiber and have been shown to regulate gut motility by activating enteric neurons and stimulating the secretion of gut hormones (Martin-Gallausiaux et al., 2021). In our study, we observed increased levels of butyrate in the feces of mice treated with *Lacticaseibacillus paracasei* DT66 and *Lactiplantibacillus plantarum* DT88, suggesting that these probiotics may influence gut motility. However, further studies are needed to examine the precise mechanisms by which SCFAs or other metabolites produced by probiotics modulate intestinal transit and contribute to the excretion of MPs. Additionally, while probiotics have been shown to alleviate inflammation in the gut by modulating the immune response (Li et al., 2022), the strain-specific effects observed in our study suggest that immune regulation could play a role in reducing the adverse effects of MP exposure. In particular, *Lactiplantibacillus plantarum* DT88 demonstrated a stronger ability to modulate inflammatory cytokine levels compared to *Lacticaseibacillus paracasei* DT66, suggesting that the anti-inflammatory properties of probiotics may be strain-dependent.

Although our study demonstrated the potential of these probiotics for MP removal in an acute model, exposure to different types of plastics, lower concentrations of MPs, larger particle sizes, as well as the long-term effects of probiotic treatment, require further investigation. Studies have shown that the harmful effects of MPs can persist with chronic low-level exposure, leading to systemic inflammation, immune dysregulation, and even organ damage (Zolotova et al., 2022). The ability of probiotics to provide long-term protection against MP-induced health risks should be evaluated in future studies, particularly focusing on their efficacy in chronic MP exposure models and their impact on overall health and gut microbiota composition.

Moreover, mechanistic insights into MP adsorption require investigating bacterial surface properties and adsorption thermodynamics. DLVO theory and thermodynamic modeling reveal that van der Waals forces, electrostatic interactions, and hydration forces govern bacterial adhesion to MPs. For instance, Liu et al. (2020) developed a plasmonic imaging technique to measure the adhesion strength of single microbial cells, providing a method to quantify interfacial forces and understand biofilm formation processes. Liu and Liu (2024) extended this approach by demonstrating that bacterial adhesion transitions from reversible to irreversible in a nanoscale, stepwise manner. Using plasmonic imaging, they showed how EPS patches act as dynamic tethers, sequentially strengthening bacterial adhesion by mediating discontinuous interactions with the substrate, a phenomenon influenced by interfacial potential energy. Additionally, Lv et al. (2022) utilized optical tracking to study surfactant-tuned bacterial adhesion, highlighting the role of surface properties in microbial attachment. These studies underscore the importance of characterizing bacterial surface properties, such as charge and hydrophobicity, in understanding and optimizing MP adsorption. Experimental approaches like zeta potential and contact angle measurements, combined with adsorption isotherm modeling, could quantify these interactions, enabling a refined understanding of how *Lacticaseibacillus paracasei* DT66 and *Lactiplantibacillus plantarum* DT88 mediate MP removal. By leveraging these insights, future work can optimize probiotic strategies to mitigate MP-associated health risks.

In conclusion, our study demonstrates that strain-specific probiotics, namely *Lacticaseibacillus paracasei* DT66 and *Lactiplantibacillus plantarum* DT88, can effectively adsorb and facilitate MP excretion, leading to up to a 67% reduction of residual MP in the mouse intestine. Furthermore, *Lactiplantibacillus plantarum* DT88 effectively mitigates intestinal inflammation caused by MP exposure in mice. Therefore, this novel probiotic-mediated MP removal strategy holds promise for addressing the health risks associated with MP exposure.

## Data Availability

The datasets presented in this study can be found in online repositories. The names of the repository/repositories and accession number(s) can be found at: https://www.ncbi.nlm.nih.gov/genbank/, OR835426.1; OR835427.1; OR835428.1.
