# Correction: Novel probiotics adsorbing and excreting microplastics *in vivo* show potential gut health benefits

**DOI:** 10.3389/fmicb.2025.1760123

**Published:** 2026-01-05

**Authors:** Frontiers Production Office

**Affiliations:** Frontiers Media SA, Lausanne, Switzerland

**Keywords:** microplastics, probiotics, *Lactobacillus*, adsorption, MP-associated health risks, high-throughput screening

File “Supplementary File 1” was erroneously published with the original version of this paper. The file has now been removed.

The correct supplementary material “Table 1” has been published.

The original version of this article has been updated.

