# Supplementary material for: Novel probiotics adsorbing and excreting microplastics in vivo show potential gut health benefits
Source: Front Microbiol. 2025 Jan 10;15:1522794. doi: 10.3389/fmicb.2024.1522794 (PMC11757873; doi:10.3389/fmicb.2024.1522794)
Supplement: Supplementary file 1 [file Table_1.docx]

Supplementary Material

Novel probiotics adsorbing and excreting microplastics in vivo shows potential gut health benefits

Xin Teng^1^, Tengxun Zhang^1^, Chitong Rao^1*^

1. Bluepha Co., Ltd., Shanghai, China

*** Correspondence:** Chitong Rao, [raochitong@gmail.com](mailto:raochitong@gmail.com)


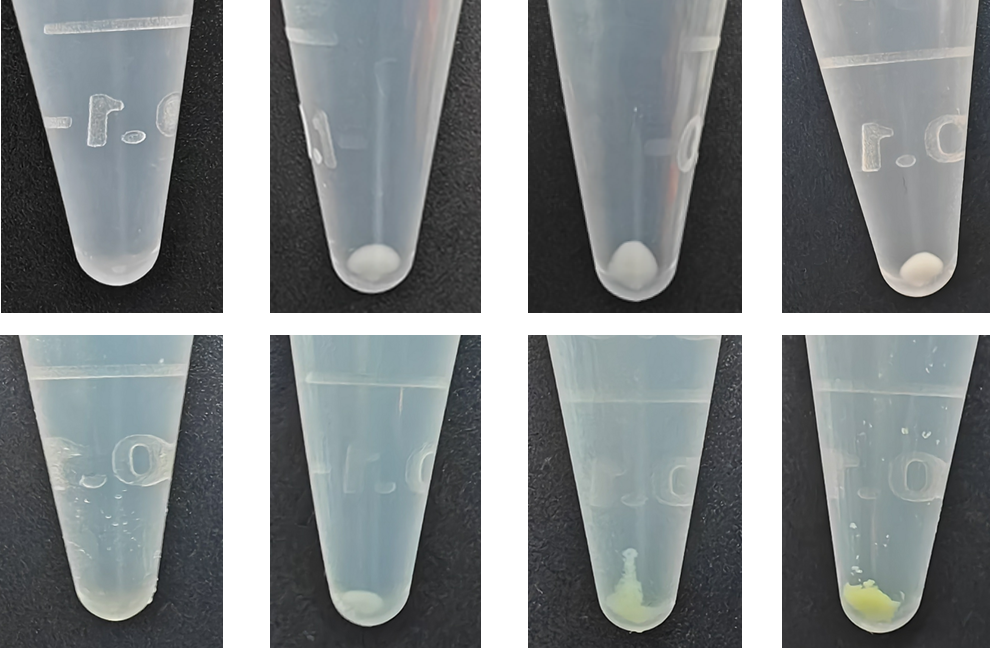


PBS

PS

Ctrl

DT22

DT66

DT88

Figure S1. Adsorption of selected Lactobacillus strains with PS fluorescent particles


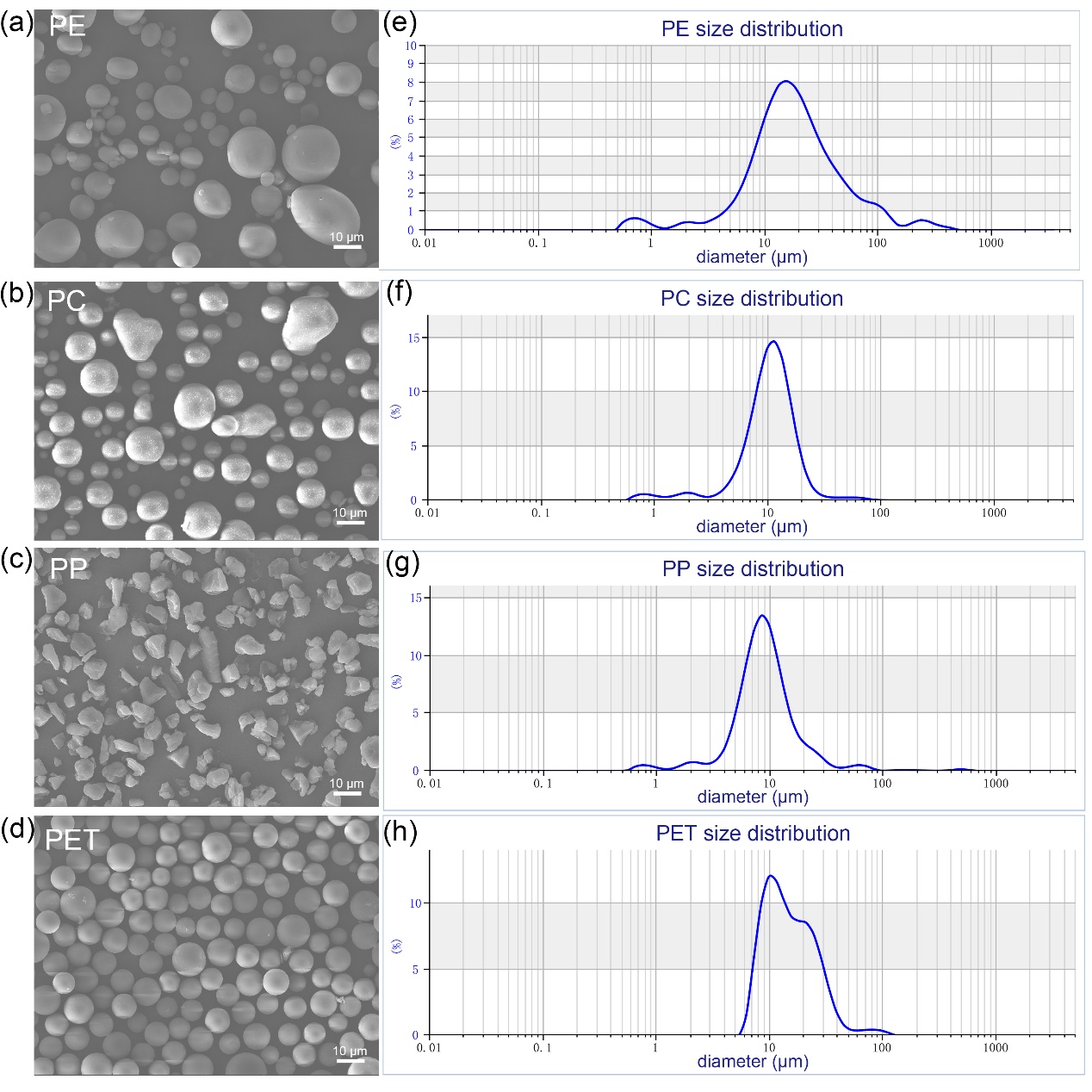


Figure S2. Representative SEM images and particle size distribution used in the study.

(a-d) Representative SEM images of PE, PC, PP and PET, with 1 000× magnification and 10 μm scale bar. (e-h) Size distribution of MP particles.


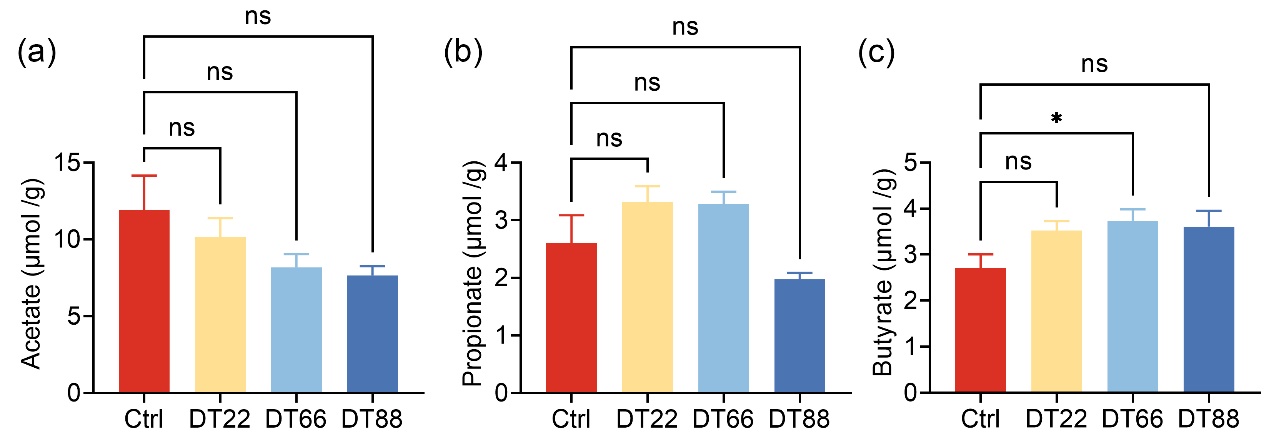


## Figure S3. SCFA concentrations in feces after 7-day probiotics gavage.

Data are shown as Mean ± SEM (μmol/g of feces), ns not significant, * p< 0.05.


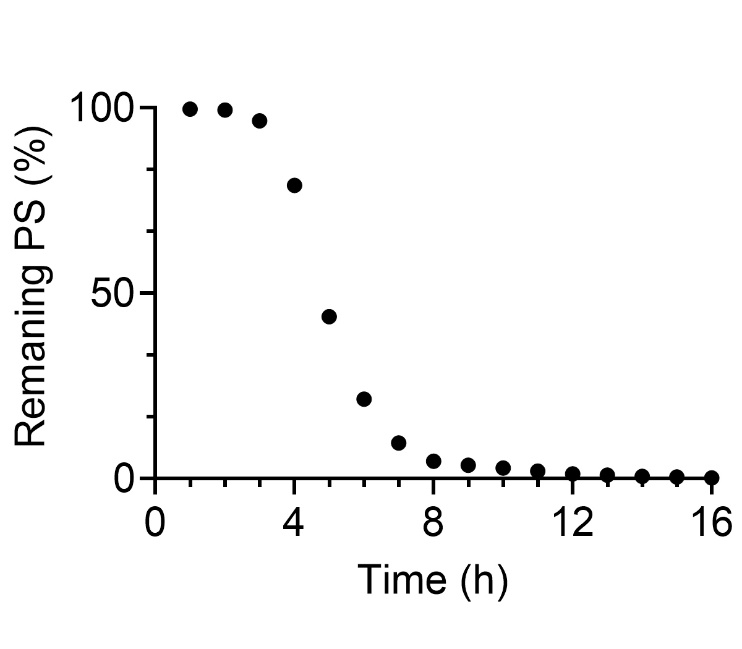


## Figure S4. The amount of PS remaining in feces sampled at different time points after a single-dose oral gavage of PS particles in mice.


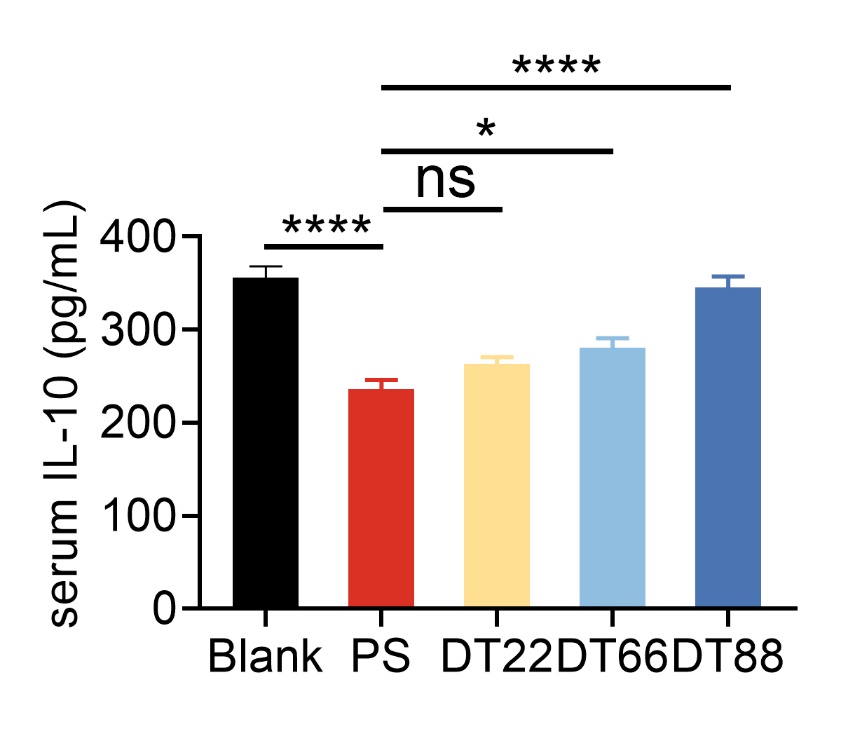


## Figure S5. Concentration of IL-10 in the serum.

Data are shown as means ± SEM, ns not significant, * p< 0.05, **** p< 0.0001.

Reference

Li, S., Zhao, Y., Zhang, L., Zhang, X., Huang, L., Li, D., Niu, C., Yang, Z., Wang, Q., 2012. Antioxidant activity of *Lactobacillus plantarum* strains isolated from traditional Chinese fermented foods. Food Chemistry 135, 1914–1919. https://doi.org/10.1016/j.foodchem.2012.06.048
